# Systems-level comparison of host responses induced by pandemic and seasonal influenza A H1N1 viruses in primary human type I-like alveolar epithelial cells *in vitro*

**DOI:** 10.1186/1465-9921-11-147

**Published:** 2010-10-28

**Authors:** Suki MY Lee, Renee WY Chan, Jennifer L Gardy, Cheuk-kin Lo, Alan DL Sihoe, Sara SR Kang, Timothy KW Cheung, Yi Guan, Michael CW Chan, Robert EW Hancock, Malik JS Peiris

**Affiliations:** 1 Department of Microbiology, The University of Hong Kong, Hong Kong SAR, PR China; 2 Department of Pathology, The University of Hong Kong, Hong Kong SAR, PR China; 3 British Columbia Centre for Disease Control, Vancouver, British Columbia, Canada; 4 Department of Cardiothoracic Surgery, Queen Elizabeth Hospital, Kowloon, Hong Kong SAR, PR China; 5 Department of Cardiothoracic Surgery, Queen Mary Hospital, Pokfulam, Hong Kong SAR, PR China; 6 Centre for Microbial Diseases and Immunity Research, University of British Columbia, Vancouver, British Columbia, Canada; 7 The University of Hong Kong-Pasteur Research Centre, Hong Kong SAR, PR China

## Abstract

**Background:**

Pandemic influenza H1N1 (pdmH1N1) virus causes mild disease in humans but occasionally leads to severe complications and even death, especially in those who are pregnant or have underlying disease. Cytokine responses induced by pdmH1N1 viruses *in vitro *are comparable to other seasonal influenza viruses suggesting the cytokine dysregulation as seen in H5N1 infection is not a feature of the pdmH1N1 virus. However a comprehensive gene expression profile of pdmH1N1 in relevant primary human cells *in vitro *has not been reported. Type I alveolar epithelial cells are a key target cell in pdmH1N1 pneumonia.

**Methods:**

We carried out a comprehensive gene expression profiling using the Affymetrix microarray platform to compare the transcriptomes of primary human alveolar type I-like alveolar epithelial cells infected with pdmH1N1 or seasonal H1N1 virus.

**Results:**

Overall, we found that most of the genes that induced by the pdmH1N1 were similarly regulated in response to seasonal H1N1 infection with respect to both trend and extent of gene expression. These commonly responsive genes were largely related to the interferon (IFN) response. Expression of the type III IFN *IL29 *was more prominent than the type I IFN *IFNβ *and a similar pattern of expression of both IFN genes was seen in pdmH1N1 and seasonal H1N1 infection. Genes that were significantly down-regulated in response to seasonal H1N1 but not in response to pdmH1N1 included the zinc finger proteins and small nucleolar RNAs. Gene Ontology (GO) and pathway over-representation analysis suggested that these genes were associated with DNA binding and transcription/translation related functions.

**Conclusions:**

Both seasonal H1N1 and pdmH1N1 trigger similar host responses including IFN-based antiviral responses and cytokine responses. Unlike the avian H5N1 virus, pdmH1N1 virus does not have an intrinsic capacity for cytokine dysregulation. The differences between pdmH1N1 and seasonal H1N1 viruses lay in the ability of seasonal H1N1 virus to down regulate zinc finger proteins and small nucleolar RNAs, which are possible viral transcriptional suppressors and eukaryotic translation initiation factors respectively. These differences may be biologically relevant and may represent better adaptation of seasonal H1N1 influenza virus to the host.

## Background

Pandemic H1N1 remains a mild disease although occasionally severe complications and death may ensue, especially in those who are pregnant or have underlying respiratory, cardiac or endocrine diseases or morbid obesity [1]. We and others have demonstrated that pdmH1N1 virus does not differ from seasonal influenza viruses in its induction of cytokine responses in human macrophages and epithelial cells [2-4]. This suggests that the cytokine dysregulation seen in H5N1 infection is not an intrinsic feature of the pdmH1N1 virus.

The pdmH1N1 virus arose from genetic reassortment between influenza viruses endemic in swine, a North American triple-reassortant swine influenza virus acquiring a neuraminidase and matrix (*M*) gene segment from viruses of the Eurasian-avian-like swine virus lineage [5,6]. Since these swine viruses have in turn originated via complex genetic reassortments between swine, avian and human influenza viruses, the pdmH1N1 virus has a novel gene constellation with virus gene segments that are derived from human (*PB1*), classical swine H1N1 (*HA, NP, NS*), Eurasian avian-like swine (*M, NA*) and avian (*PB2, PA*) sources. While the precursor swine viruses were clearly well adapted to circulate in pigs for periods ranging from 11 (North American triple reassortant) to 90 (classical swine) years, evolutionary dating analysis suggests that the pdmH1N1 virus transmitted in humans only a few months prior to its detection in March 2009 [6].

Using the Affymetrix microarray platform, we had previously demonstrated that avian H5N1 viruses elicit host responses that were qualitatively similar but quantitatively markedly different to seasonal influenza H1N1 virus in human macrophages [7]. As the tracheo-bronchial epithelium, type I and II alveolar epithelial cells and macrophages are key target cells for pdmH1N1 infection [8] and the most serious complication of pdmH1N1 disease is primary viral pneumonia, we employed type I-like alveolar epithelial cells as a model to examine the host transcriptomes induced by pdmH1N1 viruses compared with that of seasonal H1N1 viruses using the same Affymetrix microarray platform. We aimed to identify the mechanistic differences in host responses induced by these two H1N1 viruses, in order to provide insights into virus pathogenesis, which may in turn be relevant to therapeutic strategies for the treatment of influenza.

## Methods

### Viruses

The viruses used were the pdmH1N1 2009 influenza A virus (A/Hong Kong/415742/2009) and human seasonal H1N1 influenza A virus (A/Hong Kong/54/1998). From their initial isolation, the viruses were propagated in Madin-Darby canine kidney (MDCK) cells. Virus infectivity was determined by cytopathic assays on MDCK cells and quantified as 50% tissue culture infectious dose (TCID_50_). Infectious material was handled in a bio-safety level 3 facility at the Department of Microbiology, The University of Hong Kong.

### Isolation of primary human alveolar type II alveolar epithelial cells

Primary type II alveolar epithelial cells were isolated using human non-malignant lung tissue as previously described [3] obtained from patients undergoing lung resection in the Department of Cardiothoracic Surgery, Queen Mary Hospital, Hong Kong SAR, under a study approved by the Institutional Review Board of the University of Hong Kong and Hospital Authority Hong Kong West Cluster. Written informed consent was provided by each patient. Briefly, after removing visible bronchi, the lung tissue was minced into pieces of >0.5 mm thickness using a tissue chopper and washed with balanced salt solution (BSS) containing Hanks' balanced salt solution (Gibco) with 0.7 mM sodium bicarbonate (Gibco) at pH 7.4 for 3 times to partially remove macrophages and blood cells. The tissue was digested using a combination of 0.5% trypsin (Gibco) and 4 U/ml elastase (Worthington Biochemical Corporation, Lakewood, NJ, USA) for 40 min at 37°C in a shaking water-bath. The digestion was stopped by adding DMEM/F12 medium (Gibco) with 40% FBS in and DNase I (350 U/ml) (Sigma). Cell clumps were dispersed by repeatedly pipetting the cell suspension for 10 min. A disposable cell strainer (gauze size of 50 μm) (BD Science) was used to separate large undigested tissue fragments. The single cell suspension in the flow-through was centrifuged and resuspended in a 1:1 mixture of DMEM/F12 medium and small airway basal medium (SABM) (Lonza) supplemented with 0.5 ng/ml epidermal growth factor (hEGF), 500 ng/ml epinephrine, 10 μg/ml transferrin, 5 μg/ml insulin, 0.1 ng/ml retinoic acid, 6.5 ng/ml triiodothyronine, 0.5 μg/ml hydrocortisone, 30 μg/ml bovine pituitary extract and 0.5 mg/ml BSA together with 5% FBS and 350 U/ml DNase I. The cell suspension was plated on plastic flask (Corning) and incubated in a 37°C water-jacketed incubator with 5% CO_2 _supply for 90 min. The non-adherent cells were layered on a discontinuous cold Percoll density gradient (densities 1.089 and 1.040 g/ml) and centrifuged at 25×*g *for 20 min without brake. The cell layer at the interface of the two gradients was collected and washed four times with BSS to remove the Percoll. The cell suspension was incubated with magnetic beads coated with anti-CD14 antibodies at room temperature (RT) for 20 min under constant mixing. After the removal of the beads using a magnet (MACS CD14 MicroBeads), cell viability was assessed by trypan-blue exclusion. The purified alveolar epithelial cell suspension was resuspended in small airway growth medium (Lonza) supplemented with 1% FBS, 100 U/ml penicillin and 100 μg/ml streptomycin, and plated at a cell density of 3×10^5 ^cells/cm^2^. The cells were maintained in a humidified atmosphere (5% CO_2_, 37°C) under liquid-covered conditions, and growth medium was changed daily starting from 60 h after plating the cells.

### Type I-like alveolar epithelial cell differentiation

The purified type II alveolar epithelial cell pellet (passage 1 or 2) was resuspended in medium to a final concentration that allowed seeding at 5 × 10^5 ^cells/cm^2 ^onto culture flask and cultured for 14 to 20 days with the small airway culture medium SAGM (Lonza). The cells spread to form a confluent monolayer and the culture medium was changed every 48 hbefore being used for virus infection experiments.

### Virus infection of type I-like alveolar epithelial cells

Type I-like alveolar epithelial cells were infected with pdmH1N1 and seasonal H1N1 at a multiplicity of infection (MOI) of two. Minimum Essential Medium (MEM) (Gibco) with 100 U/ml penicillin and 100 μg/ml streptomycin was used as inoculum in the mock infected cells. The cells were incubated with the virus inoculum for 1 h in a water-jacketed 37°C incubator with 5% CO_2_. Then the cells were rinsed 3 times with warm PBS and replenished with the appropriate growth medium. The infected cells were harvested for mRNA collection at 8 h post-infection and viral *M *gene was quantified using real-time PCR. Total RNA was extracted from cells after 8 h post-infection using the RNeasy Mini kit (Qiagen) according to the manufacturer's recommended protocol.

### Microarray Analysis

Human gene expression was examined with the GeneChip Human Gene 1.0 ST array (Affymetrix). The Human Gene 1.0 ST array comprises more than 750,000 unique 25-mer oligonucleotide features, constituting over 28,000 gene-level probe sets. RNA quality control, sample labelling, GeneChip hybridization and data acquisition were performed at the Genome Research Centre, The University of Hong Kong. The quality of total RNA was checked by the Agilent 2100 bioanalyzer. The RNA was then amplified and labeled with GeneChip^® ^WT Sense Target Labeling and Control Reagents kit (Affymetrix). cDNA was synthesized, labeled and hybridized to the GeneChip array according to the manufacturer's protocol. The GeneChips were finally washed and stained using the GeneChip Fluidics Station 450 (Affymetrix) and then scanned with the GeneChip Scanner 3000 7G (Affymetrix).

GeneSpring GX 11 (Agilent) was used for the normalization, filtering and statistical data analysis of the Affymetrix microarray data. The linear data was first summarized using Exon Robust Multichip Average (RMA) summarization algorithm on the CORE probesets and Baseline Transformation to Median of all samples for three major tasks including Background Correction, Normalization and Probe Summarization. Briefly, Exon RMA performed a GC based background correction followed by Quantile Normalization and subsequently performed a Median Polish probe summarization. Next, quality control on samples was performed at different levels including 1) internal controls to check the RNA sample quality, 2) hybridization controls to assess the hybridization quality and 3) Principal Component Analysis (PCA) to check the data quality. Only samples that found to be satisfactory in all quality control tests were included in further analysis. In the process of data filtering, probesets with an intensity value of the lowest 20th percentile of all the intensity values were removed. The filtered entities resulted in a working transcript list used for statistical analysis. An analysis of variance (ANOVA) was performed to identify genes significantly expressed (*p *< 0.05) in response to virus infection. In order to reduce the overall false positive hits, Benjamini and Hochberg multiple testing correction was employed. Significantly differentially expressed genes with fold change ≥1.5 in response to pdmH1N1 and seasonal H1N1 infection compared with mock were then merged into a gene list for further GO and pathway analysis.

GO and pathway over-representation analysis as well as further analysis of protein-protein interactions and transcription factor regulation were carried out using the InnateDB platform [9,10]. Over-representation analyses were performed using a hypergeometric algorithm, and over-represented GO terms or pathways with *p*-values ≤ 0.05 were retained provided at least two uploaded genes mapped to the entity in question. In parallel, an independent pathway over-representation analysis was also performed using the GeneSpring program. Human pathway databases, including Integrating Network Objects with Hierachies (INOH), Reactome, Kyoto Encyclopedia Genes and Genomes (KEGG), Biocarta, National Cancer Institute (NCI) and NetPath, were imported into the software for pathway analysis of statistically significant genes.

### Real-time quantitative RT-PCR assays

Total RNA was isolated using the RNeasy Mini kit (Qiagen) as described. The cDNA was synthesized from mRNA with poly(dT) primers and Superscript III reverse transcriptase (Invitrogen). Transcript expression was monitored using a Power SYBR^® ^Green PCR master mix kit (Applied Biosystems) with corresponding primers. The fluorescence signals were measured using the 7500 real-time PCR system (Applied Biosystems). The specificity of the SYBR^® ^Green PCR signal was confirmed by melting curve analysis. The threshold cycle (CT) was defined as the fractional cycle number at which the fluorescence reached 10 times the standard deviation of the base-line (from cycle 2 to 10). The ratio change in target gene relative to the β-actin control gene was determined by the 2^-ΔΔCT ^method as described elsewhere [11].

### Microarray data accession number

Microarray data has been deposited in the Gene Expression Omnibus (GEO) database [12] with the accession number: GSE24533.

## Results

We used the Affymetrix GeneChip Human Gene 1.0 ST array to compare the global gene expression profiles of human primary type I-like alveolar epithelial cells from three independent donors (n = 3) after infection with pdmH1N1, seasonal H1N1 viruses or mock control infection at 8 h post-infection. Changes were observed in 602 transcripts from 434 individual host genes (*p *< 0.05 in one-way ANOVA test).

In a preliminary analysis, the gene expression data from each epithelial cell donor was analyzed separately to define the donor-to-donor variation after influenza infection. We used a ± 1.5-fold change in gene expression as the cut-off value and genes were classified into those that were ≥ 1.5-fold up-regulated (+) or down-regulated (-) relative to mock-infected cells and those with no change in expression (fold change between -1.5 and +1.5).

Overall, 93.2% and 74.6% of genes were concordantly expressed in the alveolar epithelial cells from the three donors after infection with pdmH1N1 and seasonal H1N1 virus respectively. The expression of those genes with discordant results among donors was further analyzed. In 36 of 41 instances (87.8%) after pdmH1N1 infection and all instances after seasonal H1N1 infection, the apparently discordant genes had the same trend of expression, being either up- or down-regulated in all donors and the differences only reflected whether the cut-off of ≥ 1.5-fold change in gene expression compared to mock-infected cells was met. The remaining five genes showed a contradictory regulation in cells from different donors infected with pdmH1N1 virus. These included *C20orf94 *(chromosome 20 open reading frame 94), *IPP *(intracistemal A particle-promoted polypeptide), *MRPL30 *(mitochondrial ribosomal protein L30), *RTN4IP1 *(reticulon 4 interacting Protein 1) and *SNORD44 *(small nucleolar RNA, C/D box 44).

**Figure 1:**
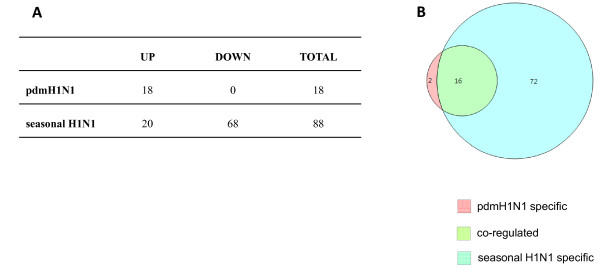
**Summary of genes expressed in response to pdmH1N1 and seasonal H1N1 infection**. (A) Genes that are significantly regulated (p < 0.05 and fold change ≥1.5) in response to pdmH1N1 and seasonal H1N1 compared with mock infection at 8h post-infection are shown. (B) Venn-diagram showing the genes that are differentially expressed in response to pdmH1N1 or seasonal H1N1 only and those that are co-regulated by both viruses.

Given the high overall concordance in gene expression profiles found among the three donors in our analysis, the fold change of gene expression levels in response to either the pdmH1N1 or seasonal H1N1 respectively, compared to mock infection, was averaged across the three donors for subsequent analysis. We filtered the average gene-expression data using a cut-off value of 1.5-fold up- or down-regulation in the pdmH1N1- and seasonal H1N1-infected cells compared to mock infected cells. Compared to mock infected cells, 88 genes were up or down-regulated in response to seasonal H1N1 infection while 18 genes were affected in pdmH1N1 infected cells, all of them being up-regulated (Figure 1A and Additional File 1: Summary of gene expression in response to influenza A virus infection).

Sixteen of the 18 genes induced by the pdmH1N1 were similarly regulated in response to seasonal H1N1 infection with respect to both trend and extent of gene expression (Figure 1B). Only two genes, basic leucine zipper transcription factor, ATF-like 2 (*BATF2*) and solute carrier family 15, member 3 (*SLC15A3*) were differentially expressed in response to pdmH1N1 infection only. On the other hand, there were 72 genes (68 genes were down-regulated and 4 genes up-regulated) affected in response to seasonal H1N1 but not in response to pdmH1N1 infection when compared with the mock infected cells (Figure 1B).

**Figure 2:**
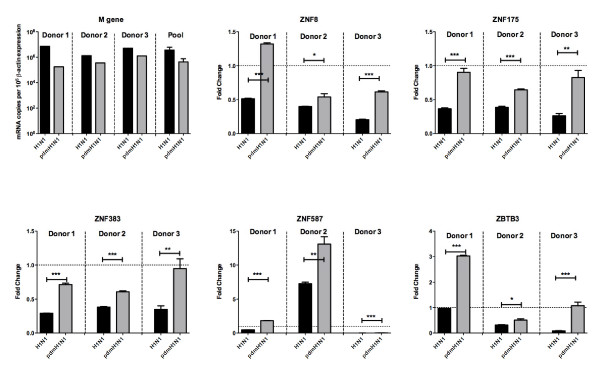
**Validation of microarray data by real-time PCR**. Expression of viral M gene and five *ZNF *genes were assessed after 8 h infection by pdmH1N1 and seasonal H1N1 viruses compared to mock. Data shown was from three individual donors denoted as donor 1, 2 and 3.

**Figure 3:**
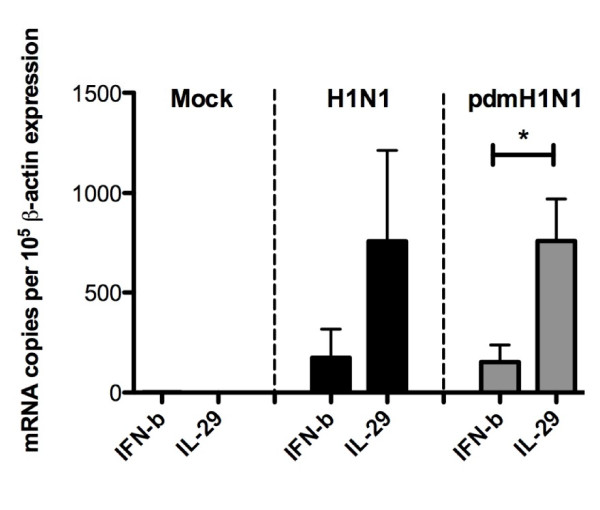
**Validation of IFN gene expression by real-time PCR**. Expression of type I (*IFNβ*) and type III (*IL29) *IFNs were assessed by real-time PCR in pdmH1N1-, seasonal H1N1- and mock infected cells at 8 h post-infection. The gene expression level averaged from the three individual donors is shown.

In order to compare the viral replication efficiency of the two viruses, the expression level of viral *M *gene was determined using real-time PCR (Figure 2). Although there was a trend to higher *M *gene copy numbers in cells infected with seasonal H1N1 virus, the differences were not statistically significant and comparable infectious viral titres were detected in the cell supernatant by viral titration. Genes of particular interest indentified in the microarray analysis were verified using real time quantitative PCR (Figures 2 and 3).

In order to investigate whether the trend towards higher virus replication with seasonal H1N1 virus was responsible for the difference in the gene expression we carried out an experiment using MOI = 6. The *M *gene expression of the two viruses was similar, but the differential expression of *ZBTB3, ZNF175, ZNF383, ZNF587 and ZNF8 *genes with expression in seasonal H1N1 infected cells being lower than pdmH1N1 infected cells was maintained.

### Over-representation analysis using InnateDB

To determine the biological relevance of the host gene expression elicited by the two viruses and in particular to identify any differences observed between these viruses, we compared the over-represented GO terms and biological pathways associated with the pdmH1N1-regulated genes to those associated with the genes altered in response to seasonal H1N1. We used the InnateDB analysis environment, and verified the results of GO and pathway analyses using GeneSpring.

**Figure 4:**
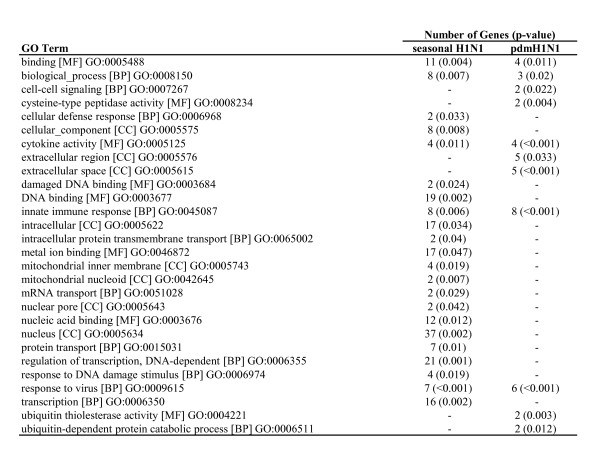
**Significantly enriched GO terms in response to seasonal and pandemic H1N1 infection**. MF = molecular function, BP = biological process, CC = cellular component. Only GO terms to which at least two differentially expressed genes were mapped are included.

We observed that host responses induced by both viruses were associated with ontological entities related to innate immunity and responses to virus infections. However, the genes expressed only in response to seasonal influenza virus were associated with DNA binding and transcription-related functions (Figure 4).

**Figure 5:**
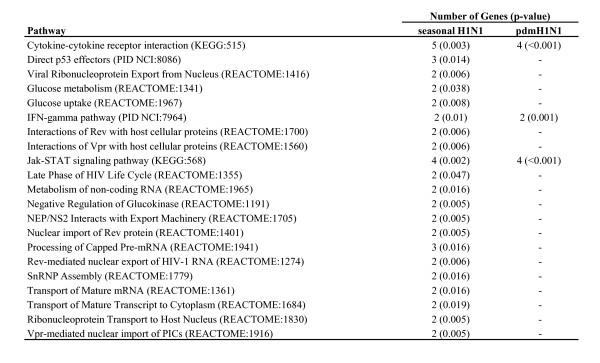
**Significantly enriched pathways in response to seasonal and pandemic H1N1 infection**. Only pathways to which at least two differentially expressed genes were mapped are included.

Pathway analysis returned a similar result, with genes regulated in response to both viruses belonging to classical innate immune response pathways, while genes regulated in response to seasonal H1N1 infection only demonstrating functions related to transcription and mRNA transport (Figure 5).

Comparison of the differentially expressed gene lists to Interferome [13,14], an IFN-regulated gene database, revealed that of the 16 genes up-regulated in response to both seasonal and pandemic H1N1 infection, 15 of these (93.75%) are related to the IFN response. A transcription factor over-representation analysis was also performed using InnateDB in order to identify transcription factors involved in the regulation of seasonal-, pandemic- and shared-response genes. Of the 13 transcription factors regulating genes affected by both seasonal and pandemic viruses, four (IRF1, IRF2, IRF7, IRF8) are known IFN response factors.

We also used InnateDB to compare the interactions between genes differentially expressed in response to either virus. Only a single difference was observed, with the seasonal H1N1 response network distinguished by the presence of the interacting DNA damage response-related genes DNA-damage-inducible transcript 4 (*DDIT4*) and RAP1 interacting factor (*RIF1*), both of which were down-regulated in response to seasonal H1N1 but unchanged in response to pdmH1N1.

## Discussion

### Comparable IFN responses to pdmH1N1 and seasonal H1N1 infection

In this study, we found that 16 out of 18 genes (88.9%) induced by the pdmH1N1 virus were also similarly regulated in response to seasonal H1N1 infection, and there was no significant difference in expression level between the two viruses.

Among these 16 genes, 15 were either IFNs or IFN-stimulated genes and we found comparable up-regulation of the type III IFNs, *IL28A, IL28B *and *IL29 *following seasonal H1N1 or pdmH1N1 infection. Although type I and type III IFNs bind to distinct receptors, they elicit similar intracellular signals and gene expression profiles [15]. *IL28A, IL28B *and *IL29 *are recognized type III IFNs which signal through a receptor complex consisting of *IL10R2 *and *IFNλR1*. Upon binding of IFNs, corresponding receptor subunits dimerize to form the receptor complex and activate the JAK-STAT signalling pathway, which then results in downstream induction of genes such as *ISGF3*, a trimetric transcription factor complex of signal transducer and activator of transcription 1 and 2 (*STAT1, STAT2*) and IFN regulatory factor 9 (*IRF9*). Downstream genes regulated by this mechanism include genes reported to have anti-viral activity such as IFN-stimulated gene 15 (*ISG15*) and myxovirus (influenza virus) resistance 1 (*MX1*) [16]. In this study, we found that IFN-related genes including *IL28A, IL28B, IL29, IRF9, ISG15 *and *MX1 *were significantly up-regulated in response to both pdmH1N1 and seasonal H1N1 infections and to a similar degree, suggesting that similar host anti-viral mechanisms are triggered in response to both H1N1 viruses.

In the microarray data, we were unable to detect the expression of type I IFNs, such as *IFNβ*, in response to either pdmH1N1 or seasonal H1N1 infection. However, we confirmed by real-time PCR the expression of *IFNβ *in response to both viruses, though present, it was notably lower when compared with type III IFN, *IL29 *(Figure 3). Similar patterns of expression of both IFN genes were seen in pdmH1N1 and seasonal H1N1 infection. This is in agreement with our previous finding that there was very low induction of type I IFNs in response to pdmH1N1 or seasonal H1N1 in alveolar epithelial cells and bronchial epithelial cells at 6 h post-infection [3], which is probably related to the potent activity of viral immune evasion genes such as *NS1*. Our results indicate that type III IFNs are likely to be particularly important in host defence in both pdmH1N1 and seasonal H1N1, possibly even more so than type I IFNs.

### Lack of host translational control by small nucleolar RNA in response to pdmH1N1 infection

When we examined genes expressed in response to seasonal H1N1 influenza virus but not pdmH1N1 virus, we noted a number of genes with roles in transcriptional or translation control, including DNA binding and mRNA transport. These genes were down-regulated in response to infection with seasonal H1N1 influenza virus relative to pdmH1N1. Several of these down-regulated genes are small nucleolar RNAs. Previous studies have suggested that host translational machinery is suppressed by the down-regulation of small nucleolar RNAs, such as *SNORA4*, in cells following influenza A infection [17]. Here we observe that *SNORA4 *is significantly down-regulated in response to seasonal H1N1 infection but not in pdmH1N1 infected cells, suggesting that seasonal H1N1 virus may be more efficient at suppressing host translational mechanisms, allowing for efficient translation of viral mRNA [18-20]. We also identified six small nucleolar RNAs that may potentially act through a similar mechanism while the functions of individual candidates in influenza pathogenesis will require further investigation.

### Lack of the control of transcriptional suppression by zinc finger proteins in pdmH1N1 infected cells

We found that nine of the genes down-regulated in response to seasonal H1N1 influenza virus (but not pdmH1N1) encode zinc finger proteins, including *ZNF175*. *ZNF175 *contains 13 zinc fingers and a KRAB domain, for a motif known to be associated with transcriptional suppression [21]. Previous data have suggested that zinc finger proteins are up-regulated in response to HIV infection and that they inhibit production of new virus through suppression of the HIV long terminal repeat (LTR) promoter activity [21]. Further work demonstrated that this suppression occurs via direct binding to two distinct regulatory regions: the negative regulatory element and the Ets element [22]. To date, no correlation between zinc finger proteins and influenza virus has been reported, however, we showed in this study that there was a significant down-regulation of multiple zinc finger proteins in response to seasonal H1N1 infection compared with pdmH1N1. Further study will be important to investigate if there is an antiviral role of these zinc finger proteins against influenza infection.

### Comparison with Transcriptomic Data from Experimental Animal Infection

Recently, a microarray analysis was reported characterizing host immune responses in ferret lung following infection with the pdmH1N1 (A/California/07/2009) and seasonal H1N1 (A/Brisbane/59/2007) [23]. In concordance with our study, they observed that IFN responses were triggered early after infection by both H1N1 viruses. However, in contrast to our data, they report that the range and magnitude of ISGs induced by seasonal H1N1 was more limited compared to pdmH1N1. However, these results are confounded by the fact that seasonal influenza replicated less efficiently in the ferret lung compared to pdmH1N1 and clearly lower levels of infection will be associated with lower induction of host responses. Thus, data from animal studies cannot differentiate whether the observed effects were due to intrinsic differences in host responses induced by the viruses or whether they reflect the viral replication competence in particular tissues in the experimental animal model used. Our data arises from a single-cycle synchronous infection of cells with an equivalent virus dose and is therefore more relevant to investigate the host responses that are driven by intrinsic differences between the two H1N1 viruses. It is also noteworthy that although seasonal influenza H1N1 replicated less efficiently than pdmH1N1 in ferret lung *in vivo*, the two viruses replicate comparably in human type I alveolar epithelial cells and in *ex vivo *lung cultures [3]. Arguably, the *ex vivo *lung data showing comparable viral tropism and replication competence with seasonal H1N1 and pdmH1N1 reflects more closely the epidemiology of the pandemic where pdmH1N1 disease severity was in fact comparable or milder than that seasonal influenza. If the differences in disease severity observed following experimental infection of ferrets was a true reflection of human disease, it would be expected that pdmH1N1 would be markedly more severe in humans than it appears to be. These observations in fact highlight the relevance of using primary and *ex vivo *human cell culture data to complement data from experimental animals.

## Conclusions

In this study, we compared the host response to seasonal and pandemic H1N1 influenza virus in a relevant human respiratory cell model, the primary human type I-like epithelial cells that are a primary target in the lung that may lead to primary viral pneumonia [24,25], including infection with the recently identified pdmH1N1 virus [3,8].

We conclude that both seasonal H1N1 and pdmH1N1 trigger similar host IFN-related antiviral responses. Type III IFNs, were more prominently induced by both viruses when compared with type I IFNs. This highlights the significance of type III IFN signalling in the pathogenesis of both pdmH1N1 and seasonal H1N1 viruses. In agreement with our other recent findings, we observed that the cytokine and overall host response profile triggered by both viruses were similar [3,26] and that pdmH1N1 does not produce the cytokine dysregulation as seen in H5N1 infection. The difference between the pandemic and seasonal H1N1 viruses lay in their ability to potentially alter host transcriptional and translational responses. Down-regulation of zinc finger proteins and small nucleolar RNAs - possible viral transcriptional suppressors and eukaryotic translation initiation factors, respectively - may facilitate the efficient replication of seasonal H1N1 influenza virus in the host. Lacking suppression via these mechanisms suggests pdmH1N1 virus may be relatively less adapted for replication in human type I-like alveolar epithelial cells.

We demonstrate differences in regulation of ten zinc finger proteins and seven small nucleolar RNAs in host responses to pdmH1N1 and seasonal H1N1 influenza virus. The role of these proteins in influenza pathogenesis merits further investigation.

## Competing interests

The authors declare that they have no competing interests.

## Authors' contributions

SMYL, RWYC, MCWC and JSMP conceived and designed the experiments. RWYC, MCWC and SSRK generated the type I-like alveolar epithelial cells and performed the virus infection experiments in bio-safety level 3 facility. SMYL, JLG, RWYC, MCWC, TKWC, YG, REWH, JSMP analyzed the data. CKL, ADLS and REWH contributed cells, reagents and analysis tools for this study. SMYL, JLG, JSMP wrote the paper and all authors contributed to critical revision of the manuscript.

## Supplementary Material

Additional file 1 **Summary of gene expression in response to influenza A virus infection**. Fold change of gene expression in response to pdmH1N1 and seasonal H1N1 at 8 h post-infection time in human type I-like alveolar epithelial cells that showed significant difference (*p < 0.05*, with Benjamini-Hochberg multiple testing correction and fold change ≥ 1.5) in expression level compared to mock infected cells were shown. The "-" and no sign before the number indicates the down- and up-regulation of the gene respectively in influenza A infected cells compared to mock. HGNC Gene Symbol is HUGO Gene Nomenclature Committee approved gene symbol. *Ratio [pdmH1N1]/[seasonal H1N1] indicates the fold change of gene expression in response to pdmH1N1 compared to seasonal H1N1 infection at 8 h post-infection time. Click here for file
